# Multi-Faceted Intervention to Improve the Antibiotic Prescriptions among Doctors for Acute URI and Acute Diarrhoea Cases: The Green Zone Antibiotic Project

**DOI:** 10.21315/mjms2019.26.4.12

**Published:** 2019-08-29

**Authors:** Kim Heng Tay, Farnaza Ariffin, Benedict Lh Sim, Sheau Yin Chin, Ammar Che sobry

**Affiliations:** 1 Infectious Disease Unit, Medical Department, Hospital Sungai Buloh, Ministry of Health Malaysia, Sungai Buloh, Selangor, Malaysia; 2 Primary Care Medicine, Faculty of Medicine, Universiti Teknologi MARA, Sungai Buloh Campus, Selangor, Malaysia; 3 Paediatrics Department, Hospital Sungai Buloh, Ministry of Health Malaysia, Sungai Buloh, Selangor, Malaysia; 4 Emergency Department, Hospital Sungai Buloh, Ministry of Health Malaysia, Sungai Buloh, Selangor, Malaysia

**Keywords:** antibiotics, acute diarrhoea, acute upper respiratory infection, clinical audit, prescription, multifaceted intervention, rational

## Abstract

**Background:**

Antimicrobial resistance is a global problem that is perpetuated by the inappropriate use of antibiotics among doctors. This study aims to assess the antibiotic prescription rate for patients with acute upper respiratory infection (URI) and acute diarrhoea.

**Methods:**

A completed clinical audit cycle was conducted in 2018 in the busy emergency department of a public hospital in Malaysia. Pre- and post-intervention antibiotic prescription data were collected, and changes were implemented through a multifaceted intervention similar to Thailand’s Antibiotics Smart Use programme.

**Results:**

Data from a total of 1,334 pre-intervention and 1,196 post-intervention patients were collected from the hospital’s electronic medical records. The mean (SD) age of participants was 19.88 (17.994) years. The pre-intervention antibiotic prescription rate was 11.2% for acute diarrhoea and 29.1% for acute URI, both of which are above the average national rates. These antibiotic prescription rates significantly reduced post-intervention to 6.2% and 13.7%, respectively, falling below national averages. Antibiotic prescription rate was highest for young children. There were no significant changes in rates of re-attendance or hospital admission following the intervention.

**Conclusion:**

The multifaceted intervention, which included continuing medical education, physician reminders and patient awareness, was effective in improving the antibiotic prescription rates for these two conditions.

## Introduction

‘The thoughtless person playing with penicillin treatment is morally responsible for the death of the man who succumbs to infection with the penicillin-resistant organism.’ This was a warning from Sir Alexander Fleming, the physician who discovered the first antibiotic. Eighty years later, the world continues to battle antimicrobial resistance, which threatens to increase economic burden, hospitalisation and mortality (1). Globally, at least 700,000 people die each year of drug-resistant bacterial infections (2). Antibiotic overprescribing continues to be a problem, especially in self-limiting illnesses—such as acute upper respiratory illness (URI) and acute diarrhoea—that are predominantly viral in origin (3).

A systematic review reported that individuals prescribed antibiotics for a respiratory or urinary infection may develop bacterial resistance to that antibiotic, with greatest effect in the month immediately after treatment, and that resistance may persist for up to 12 months (4). These findings imply that the best way to avoid the vicious cycle of antimicrobial resistance (which can lead to the use of more powerful broad-spectrum antibiotics) is to avoid initial use whenever possible.

South East Asia is rampant with inappropriate antibiotic use, contributing immensely to antimicrobial resistance (5–7). Since 2010, South East Asian countries have been conducting national situational analyses of medicine management supported by the World Health Organization (WHO) to monitor antibiotic use in the region and to create awareness of inappropriate use (5, 8). Clinical audits to monitor antibiotic use is an important aspect of improving quality and promoting the rational use of antibiotics (9).

In Malaysia, despite the steps taken by the Ministry of Health (MoH) to promote rational use of antibiotics through published guidelines, antibiotic prescription rates remain high in hospitals and primary care settings (10, 11). Other local studies have found that antibiotic therapy remains high, especially for acute URI and acute gastroenteritis, which account for 49.2% and 20.5% of antibiotic prescriptions, respectively (12). It is concluded that passive dissemination of guidelines alone is unlikely to lead to positive change in antibiotic prescribing habits. A systematic review found that continuing medical education (CME) using multifaceted interventions that include interactive educational meetings and reminders for doctors, however, has improved antibiotic prescription rates (10, 13).

The Antibiotic Smart Use (ASU) programme, introduced in Thailand in 2007, aims to improve antibiotic prescribing by targeting both physicians and patients with acute URI and acute diarrhoea (14). One study suggests that the ASU programme’s multifaceted intervention is very effective in promoting the rational use of antibiotics for outpatient care and in reducing the antibiotic prescription rates for both these conditions (15). Following the programme’s success in improving doctors’ and patients’ rational use of antibiotics, the Green Zone Antibiotics Project (GZAP) was formed. The aim of this study was to assess and compare the antibiotic prescription rates for patients with acute URI and acute diarrhoea in a green zone emergency department (ED) hospital setting and to implement changes based on a multifaceted intervention to complete the audit cycle.

## Methodology

GZAP was a clinical audit project and collaboration among infectious disease (ID), emergency medicine, paediatric, pharmacy and primary care medical departments. It was designed to clinically audit the antibiotic prescription rates for patients with acute URI and acute diarrhoea in a green zone ED. The Malaysian hospital ED is divided into red, yellow and green zones. The red zone is for critical cases, yellow for semi-critical cases and green for non-critical cases. The study was conducted at a busy hospital serving a population of 466,163 people according to the 2010 census.

The GZAP investigators formulated a clinical audit protocol. A clinical audit is a quality improvement process that seeks to improve patient care through an assessment of the structure, process and outcome of care and the implementation of change. URI is characterised by the symptoms of sore throat, cough and rhinorrhea with or without fever. Acute diarrhoea is defined as having three or more loose or watery stools per day over a period of less than two weeks. The studied hospital’s ED has good structures in place to audit and implement changes through a multifaceted intervention. It has a working electronic medical record (EMR) system called the e-His system, which contains the patient registry and ICD-10 codes. There are dedicated physician consultation rooms with desktop computers, and there are patient information boards and multimedia screens in the patient waiting area. The hospital’s ED also has a viable process of care that includes triaging, patient registration, nurse pre-assessment and doctor consultation. The doctor then enters each patient’s diagnosis according to ICD-10 coding, and treatment given is recorded in the EMR.

GZAP was conducted using a universal sampling process over a three-month period for both pre- and post-intervention data collection. All patients aged 2 or more years who visited the green zone ED for either acute URI or acute diarrhoea during the stated data collection period were included. Data collected included patient’s unique ID number, age, sex and antibiotic prescribing outcome, including the status of the prescription, type of antibiotic prescribed and patient re-attendance to the ED within a week of presentation. Throat swab and stool culture results were not available because these are not common clinical practices within the hospital.

During the pre-intervention phase, data on patients with diagnoses of acute URI or acute diarrhoea who had attended the green zone ED between January 2018 and March 2018 were collected via the e-His system. Data with the following ICD-10 codes were collected: J00, J01.0, J01.2, J01.3, J01.4, J01.8, J01.9, J02.0, J02.8, J02.9, J03.0, J03.8, J03.9, J04.0, J04.1, J04.2, J05.0, J05.1, J06.1, J06.9, J03.0, J03.8, J03.9, J04.0, J04.1, J04.1, J04.2, J05.0, J05.1, J06.1, J06.9, J20, A000, A00, A00.9, a01.10, A01.0, A01.2, A01.3, A01.4, A02.9, A02.2, A02.8, A02.9, A03.0, A03.1, A03.2, A03.3, A03.8, A03.9, A04.0, A04.1, A04.2, A04.3, A04.4, A04.5, A04.6, A04.7, A04.8, A04.9, a05.0, A05.1, A05.2, A05.3, A05.4, A05.8, A05.9, 06.0, A06.1, A06.2, A07.1, A07.2, A07.3, A07.8 A08.0, A08.2, A08.3, A08.4, A09, K52.1, K52.3, K52.8, K52.9.

Following the first phase of data collection, changes were implemented through a multifaceted intervention to educate both physicians and patients attending the green zone ED on the rational use of antibiotics for URI and acute diarrhoea. The intervention was conducted in June and July 2018. The educational toolkits included a training module for healthcare providers on URI and acute diarrhoea in children and adults. The training module was a compulsory 1-h CME session for all physicians working in the ED and covered diagnostic criteria and decision making on antibiotic prescribing. The supplementary toolkits included patient information leaflets in both Malay and English, calendar flipcharts and laminated quick-reference guides on the clinical pathways of acute URI and acute diarrhoea, educational posters in Malay and English in the patient waiting area and doctor consultation rooms, sticky reminders at each doctor’s computer desktop and educational multimedia videos in the patient waiting area.

Following the intervention, a re-audit was conducted to complete the clinical audit cycle and data on patients with diagnoses of acute URI and acute diarrhoea who had attended the green zone ED in August and September 2018 were again collected via the e-His system.

The pre- and post-intervention data were entered and analysed using SPSS v24. Descriptive data analysis was conducted as well as Chi-squared tests. The clinical indicator is the percentage of all patients diagnosed with acute URI and acute diarrhoea who were prescribed antibiotics. It was calculated using the following formula: antibiotic prescription rate (%) = total number of patients with antibiotics prescribed for certain disease (X)/total number of patients diagnosed with disease X. Based on the data from the 2014 Malaysian National Medical Care Survey (NMCS), the standard antibiotic prescription rate in Malaysia for acute URI is less than 16.8% and for acute diarrhoea less than 9.1% (12).

Permission to publish the data was obtained from the Director General of Health through the National Institute of Health with the referenced letter KKM.NIHSEC.880-4/4/1 Jld. 67 (09).

## Results

Data from a total of 1,334 pre-intervention and 1,196 post-intervention patients with acute URI and acute diarrhoea who attended the green zone ED during the audit period were collected. The mean (SD) age of participants was 19.88 (17.994) years.

Table 1 shows the percentage of acute diarrhoea and acute URI diagnoses as well as patients’ sex and age group details. The results show that patients more commonly present with acute URI than acute diarrhoea and that children between the ages of 2 and 12 comprise half of the ED’s presentation of these conditions.

Table 2 shows that children between the ages of 2 and 12 present with acute diarrhoea or acute URI more than the other age group. The second most common age group is 20 to 39. In the pre-intervention group, the antibiotic prescription rates were high, above the national averages for both conditions. These rates reduced to below national averages in the post-intervention group. Table 2 also highlights that the most common antibiotic prescribed for acute diarrhoea during pre-intervention was amoxicillin, and the most common antibiotic prescribed during post-intervention was metronidazole. The most common antibiotic prescribed in both pre- and post-intervention for acute URI was amoxicillin, followed by erythromycin. The rate of repeat visits was similar pre- and post-intervention.

Table 3 shows a marked significant difference in antibiotic prescription rate following the multifaceted intervention. However, there is no significant difference in repeat patient visits to the ED.

## Discussion

This study found that pre-intervention antibiotic prescription rates were high, above the NMCS standards for both acute diarrhoea and acute URI, and that they reduced significantly in the post-intervention period to below the NMCS standards. Previous studies have identified various factors related to high rates of antibiotic prescribing, which include regulation and enforcement laxity, diagnostic uncertainty, perceived demand and patient expectations, practice sustainability and financial considerations, influence from medical representatives and inadequate knowledge (16, 17). Patient expectations and pressure from parents have an enormous influence on physicians’ antibiotic prescribing habits (18, 19). The improvement in post-intervention antibiotic prescription rates suggests that improving doctors’ and patients’ awareness of antibiotic use and providing continual reminders can overcome some of these factors.

It is well known that acute URI and acute diarrhoea are common among children. This was reiterated in this study, which found that the highest antibiotic prescription rate is for young children. The challenge lies in diagnostic accuracy and confident decision making to refrain from prescribing antibiotics when there are no indications for them. One factor that has been shown to influence this decision making is parent expectations, which can pressure physicians to prescribe even when they feel it is unnecessary (20). A systematic review found that physicians are rarely certain about whether to prescribe antibiotics to children and that physicians’ fear of potential poor health outcomes or legal consequences create a dilemma (20). This study found that multifaceted intervention is useful in promoting rational use of antibiotics and in reducing the antibiotic prescription rate for children. Other successful measures include delayed prescription and specific instructions to patients about when to use antibiotics (21, 22)

The appropriateness of the type of antibiotic prescribed is also important in preventing antimicrobial resistance. The golden age of antibiotics extended from their discovery in 1941 through the 1990s. During this time, any antibiotic could treat all bacterial infections, including those in critically ill patients (24). However, bacterial resistance increased, as did the realisation that prolonged use of antibiotics—especially broad-spectrum ones—can destroy the body’s normal flora, making an individual more susceptible to opportunistic infections (23).

Since then, the WHO and various countries have taken measures to reduce antimicrobial resistance. One such measure is to target appropriate antibiotics to specific conditions. This study shows that the two most common antibiotics prescribed for acute URI are amoxicillin and erythromycin. The national antibiotics guidelines (24) advocate the use of phenoxymethylpenicillin for bacterial pharyngitis and tonsillitis with amoxicillin as a second-line treatment. Amoxicillin is also used for acute rhinosinusitis and community-acquired pneumonia. Erythromycin is recommended if there is a history of penicillin allergy. These antibiotics are appropriate if a bacterial cause is suspected.

Choosing an antibiotic for acute diarrhoea is more complex and requires confirmation of the causative organism. The most commonly prescribed antibiotics for acute diarrhoea in this study were amoxicillin and metronidazole. Metronidazole is recommended for parasitic or nosocomial infections and is not recommended for most bacterial causes. The CME session conducted in this study addressed how to choose appropriate antibiotics and the pathways for acute diarrhoea and acute URI, providing physicians with a quick reference guide. Essential to antibiotic policy is the development of a restrictive list of antibiotics within a standard and regularly updated treatment guide. The MoH has produced national antibiotic guidelines (24), which are freely available to all healthcare personnel and are reviewed every 2 years to 4 years. The use of these guidelines was emphasised during the doctors’ CME training. All these measures, as well as repeated clinical audits, prescription feedback and continual reminders for physicians, are essential to developing an effective antibiotic stewardship programme.

Most antibiotic stewardship programmes have been developed in hospitals in resource rich countries. The have been found to decrease antibiotic use by 20%–40% and thus should be expanded to developing countries and within primary care settings (22). Their goals include improved patient outcomes, containment of antibiotic resistance and increased cost-effectiveness of care. The Antibiotics Smart Use (ASU) programme was initiated in Thailand in 2007 and has since been adopted as a pay-for-performance criterion and expanded nationwide (14). One study finds that the multifaceted interventions implemented following the ASU recommendations are very effective, decreasing prescription rates from 74% to 13% in acute URI and from 78% to 19.1% in acute diarrhoea (15). Malaysia’s MoH has outlined a protocol for an antibiotic stewardship programme (25), but its implementation is only slowly gaining momentum within public hospitals. This study has shown that educating physicians and the public through a multifaceted intervention can reduce antibiotic prescription rates without compromising patient outcome. Another Malaysian study similarly shows that physicians exposed to multifaceted interventions have lower antibiotic prescription rates than those who are not (26). CME and continual reminders are essential to managing antimicrobial resistance, and this training should start in medical school. One study reports that while medical students had average confidence in their ability to diagnose and choose appropriate antibiotics, they felt that they needed more training in the rational use of antibiotics (27).

## Strengths and Limitations

One strength of this study is its large sample sizes both pre- and post-intervention. Its limitations include its single study site—one public hospital in Gombak. Therefore, the results from this clinical audit may not generalise beyond this local setting. However, the effectiveness of the multifaceted intervention in reducing antibiotic prescription rates is an important finding, as the intervention can be applied to other public and private hospitals as well as primary care settings.

## Conclusion and Recommendations

In conclusion, the battle against antimicrobial resistance is ongoing, and measures such as regular clinical audits on antibiotic prescribing and multifaceted interventions to educate physicians and patients are required to promote the rational use of antibiotics. The most important problems identified by GZAP are the sustainability of such programmes and the expansion of multifaceted interventions to all public and private hospitals as well as primary care services. Other studies have identified some barriers to implementation, including insufficient leadership, time, commitment and resources (23). Time constraints proved a definite challenge during the GZAP intervention, as it was difficult to gather all the doctors to conduct a CME. Therefore, other methods of training, such as an online course that includes published slides and videos based on this study, are being developed. This online course will be freely available to all doctors.

## Figures and Tables

**Table 1:** Frequency of the diagnosis and patients details (sex and age group) among pre-intervention and post-intervention patients

	Data population	Pre-intervention (*N* = 1334) *n* (%)	Post-intervention (*N* = 1196) *n* (%)
**Diagnosis**	Acute Diarrhoea	216 (16.2)	321 (26.8)
	Acute URI	1118 (83.8)	875 (73.2)
**Sex**	Male	689 (51.6)	589 (49.2)
	Female	645 (48.4)	607 (50.8)
**Age group**	2–12	752 (56.4)	545 (45.6)
	13–19	120 (9.0)	107 (8.9)
	20–39	268 (20.1)	344 (28.8)
	40–59	134 (10.0)	151 (12.6)
	> 59	60 (4.5)	49 (4.1)

**Table 2:** Comparing pre and post intervention on the number of patients according to age group, antibiotics prescribing rate to national standard, types of antibiotics prescribed and repeated visit to ED among patients with acute diarrhoea and acute URI

No. of patients	Condition population	Pre-intervention		Post-intervention	
	
		Acute diarrhoea *N* = 216 *n* (%)	Acute URI *N* = 1118 *n* (%)	Acute diarrhoea *N* = 321 *n* (%)	Acute URI *N* = 875 *n* (%)
Total variable		*n* = 216	*n* = 1118	*n* = 321	*n* = 875
Age group	2–12	122 (56.5)	630 (56.4)	132 (41.1)	413 (47.2)
	13–19	20 (9.3)	100 (8.9)	38 (11.8)	69 (7.9)
	20–39	50 (23.1)	218 (19.5)	106 (33.0)	238 (28.2)
	40–59	13 (6.0)	121 (10.8)	33 (10.3)	118 (13.5)
	> 59	11 (5.1)	49 (4.4)	12 (3.7)	37 (4.2)
Total variable		*n* = 215*	*n* = 1117*	*n* = 321	*n* = 875
Antibiotics prescribed to patients	No	191 (88.8)	792 (70.9)	301 (93.8)	755 (86.3)
	Yes	24 (11.2)	325 (29.1)	20 (6.2)	120 (13.7)
Antibiotics prescribing rate	Rate (%)	11.2%	29.1%	6.2%	13.7%
Standard rate of antibiotics prescribed according to NMCS.	Rate (%)	9.1%	16.8%	9.1%	16.8%
Comparing antibiotics prescribing rate to the standard of NMCS	Higher or lower than the national standard	Higher	Higher	Lower	Lower
Total variable		*n* = 24	*n* = 324*	*n* = 20	*n* = 119*
Types of antibiotics used	Amoxycillin	14 (58.3)	280 (86.4)	6 (30.0)	102 (85.7)
	Erythromycin	–	32 (9.9)	–	12 (10.1)
	Cloxacillin	–	6 (1.9)	–	2 (1.7)
	Penicillin	1 (4.2)	3 (0.9)	–	1 (0.8)
	Metronidazole	8 (33.3)	–	10 (50.0)	–
	Others	1 (4.2)	3 (0.9)	4 (20.0)	2 (1.7)
Total variable		*n* = 154*	*n* = 861*	*n* = 321	*n* = 875
Repeated visits	Yes	18 (11.7)	91 (10.6)	18 (5.6)	79 (9.0)
	No	136 (88.3)	770 (89.4)	303 (94.4)	796 (91.0)

* refers to missing data

**Table 3:** Statistical analysis using Chi-squared test to identify the significance between pre-intervention and post-intervention outcomes on antibiotics prescribing rate and repeated visits

		Pre-intervention (*N* = 1015)*		Post-intervention (*N* = 1196)		Chi-squared test
	
		Number (*n*)	Rate (%)	Number (*n*)	Rate (%)	
Repeated visits	Yes	109	10.7	97	8.1	0.021
	No	906	89.3	1099	91.9	
Antibiotics prescribing	Yes	349	26.2	140	11.7	0.001
	No	983	73.7	1056	88.3	

* refers to missing data
